# Neuromuscular Electrical Stimulation to Immobilized Lower Extremities Directly Following Orthopaedic Surgery in Three Children with Cerebral Palsy: A Case Series

**DOI:** 10.3390/s21227661

**Published:** 2021-11-18

**Authors:** Kelly Greve, Caroline Colvin

**Affiliations:** Division of Occupational Therapy and Physical Therapy, Cincinnati Children’s Hospital Medical Center, Cincinnati, OH 45229, USA; caroline.colvin@cchmc.org

**Keywords:** neuromuscular electrical stimulation, cerebral palsy, immobilization, rehabilitation, physical therapy

## Abstract

Cerebral palsy (CP) is a non-progressive, neurological disorder often resulting in secondary musculoskeletal impairments affecting alignment and function which can result in orthopaedic surgery. Neuromuscular electrical stimulation (NMES) is a modality that can be used for rehabilitation; however, NMES immediately following orthopaedic surgery in children with CP using surface electrodes has not been previously reported. The purpose of this case series is to describe the novel use of NMES in the acute rehabilitation phase directly after orthopaedic surgery. This case series included three children with spastic diplegia CP, Gross Motor Function Classification System level II who underwent Single Event Multi-Level orthopaedic Surgery. Each long leg cast contained window cast cut-outs to allow for surface electrode placement for daily NMES intervention to the quadriceps muscles while immobilized. Children were assessed pre- and post-operatively using the Functional Mobility Scale (FMS), Gross Motor Function Measure (GMFM-66), and 6-Minute Walk Test (6MWT). All children demonstrated no adverse effects using NMES intervention and had improvements in the 6MWT. Most children demonstrated gains in the FMS and GMFM-66. Use of NMES through window cast-cuts in a long leg cast is a novel practice approach for delivery of early rehabilitation following lower extremity orthopaedic surgery.

## 1. Introduction

Cerebral palsy (CP) is a non-progressive lesion in the brain occurring in children under the age of two years resulting in neurological and musculoskeletal deficits affecting body movement and posture [1,2,3]. The incidence of CP in the United States is approximately 3.5 per 1000 births [4]. Cerebral palsy is often classified by muscle tone type, limb distribution [5], and gross motor function [6].

As a child with CP grows, secondary neuromotor and musculoskeletal deficits, including muscle weakness, decreased muscle length, altered muscle tone and stretch reflexes, and impaired selective motor control, can affect alignment and function [1]. Orthopaedic surgery is commonly recommended to correct soft tissue and bony deformities limiting posture and function [7]. Following surgery, children’s extremities are often immobilized contributing to muscle atrophy and weakness. As a result, physical therapy is a recommended intervention following Single Event Multi-Level orthopaedic Surgery (SEMLS) [8] to facilitate greater functional mobility [9]. 

Neuromuscular electrical stimulation (NMES) is a modality to improve motor performance and muscle recruitment of the neuromuscular system [10]. Studies have been reported using NMES in both adults and children. Early use of NMES following orthopaedic surgery in adults has been successful with improving strength and functional outcomes at one year post-operatively [11,12]. For example, adults who underwent total knee arthroplasty (TKA) have used NMES acutely after surgery to re-educate muscle activation, reduce quadriceps muscle atrophy, and facilitate muscle function recovery [11,12]. 

Neuromuscular electrical stimulation as a physical therapy intervention has a low level of evidence in the pediatric population for improving strength and gait in individuals with CP [8]. Previously, NMES has been used as an adjunct intervention to post-operative therapy for individuals with CP [13,14]. Few studies have reported using NMES following orthopaedic surgery in children with CP. Johnston et al. [13] reported surgically implanting electrodes into multiple lower extremity (LE) muscle groups in children with CP following surgery involving multiple orthopaedic procedures. Significant gains were reported in range of motion (ROM) and positive trends for improving step length, cadence, and walking velocity from baseline to four months post-operatively [13]. Results at 12 months post-operatively indicated no differences in outcomes when compared to a surgery-only group [13]. Infection, skin irritation, and electrode failure were complications noted in this study using percutaneous intramuscular implanted electrodes [13].

Standard methods and parameters for clinical use of NMES to improve function are emerging. Neuromuscular electrical stimulation directly following SEMLS in children with CP using surface electrodes as sensors to contract skeletal muscle has not been previously reported. The purpose of this case series is to describe the novel use of NMES in the acute rehabilitation phase directly after orthopaedic surgery.

## 2. Materials and Methods

### 2.1. Population

This case series included three children with spastic diplegia CP, Gross Motor Function Classification System (GMFCS) level II who underwent SEMLS. The children were males, ages 9 (Child 1), 13 (Child 2), and 15 (Child 3) years old (Table 1). Child 3 had a comorbidity of Autism. Pre-operatively, a physical therapy examination and 3D gait analysis indicated LE impairments in ROM, strength, distal selective motor control, tone, posture, and gait for all three children. Based on the results of these assessments, orthopaedic surgery was recommended for each child. All children underwent SEMLS between November and December 2019. Surgical procedures consisted of the following: Child 1: Bilateral femoral hemi-epiphysiodesis, hamstring lengthenings, adductor lengthenings, and gastrocnemius lengthenings; Child 2: Bilateral femoral hemi-epiphysiodesis, hamstring lengthenings, OnabotulinumtoxinA (BOTOX) injections to the hamstrings; and Child 3: Bilateral hamstring transfers, left posterior tibial lengthening, and OnabotulinumtoxinA (BOTOX) injections to bilateral hamstrings. Following surgery, long leg extension casts were applied in the operating room, and each child was admitted to an acute inpatient unit at a Midwestern pediatric research hospital for a brief inpatient stay. Each child was weight-bearing as tolerated immediately following surgery.

### 2.2. Intervention

Prior to surgery, each child trialed NMES and agreed to use it for quadriceps stimulation directly following orthopaedic surgery. Optimal electrode placement was determined for bilateral quadriceps contraction, and each child’s parent took photographs for future reference. The EMSI-Flex (EMSI/TMR, Tampa, FL, USA), an electrical stimulation unit with capabilities for NMES, was issued to each child. Each NMES unit was pre-programmed with parameters [14] (Table 2) to obtain a motoric quadriceps contraction. Each child and caregiver were instructed on electrode application and demonstrated independence with turning on the NMES unit to achieve optimal stimulation for quadriceps contraction. The children and caregivers were instructed to use the NMES device to practice eliciting quadriceps contraction prior to surgery. In addition, each child and caregiver were instructed to look for signs and symptoms of intolerance to the electrodes and the NMES intervention.

**Figure 1:**
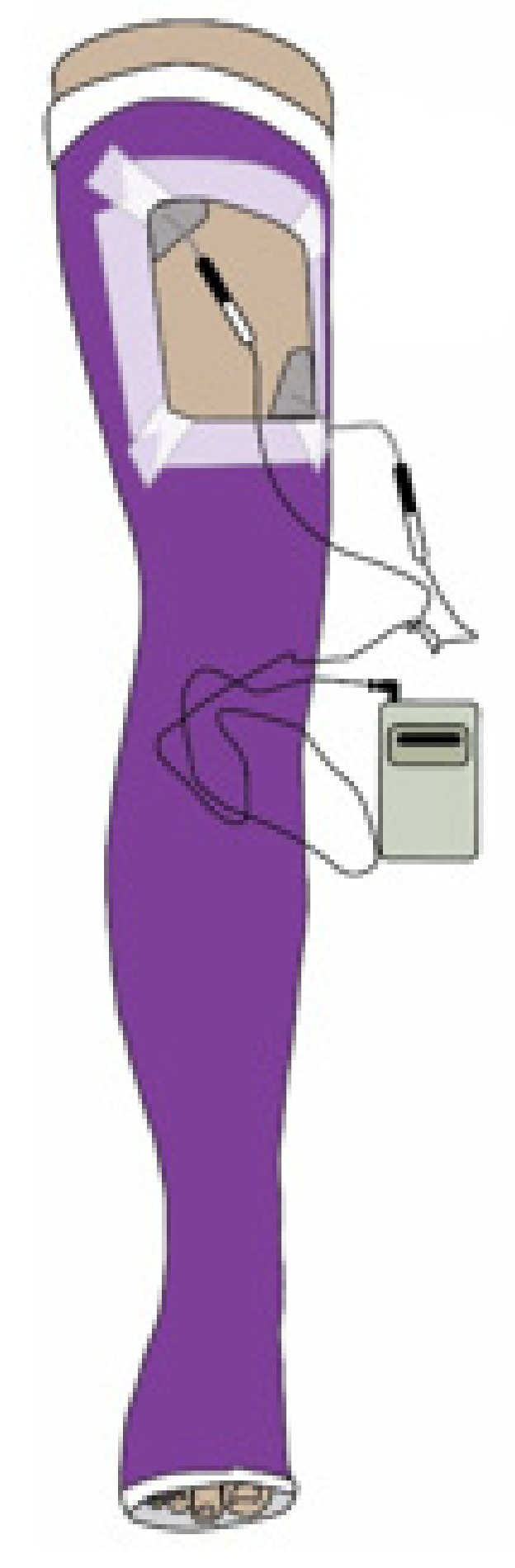
Example of window cast cut-out for surface electrodes to be placed for neuromuscular electrical stimulation intervention.

Approximately one week following surgery, window cast cut-outs (Figure 1) in the long leg casts were completed by an orthopaedic casting technician in an outpatient clinic setting. The cast-cut out locations were based on photographs from pre-operative surface electrode placement. Each child was then instructed to complete daily NMES for bilateral quadriceps strengthening for 15–30 min intervals. During the NMES intervention, the children were encouraged to perform an isometric quadriceps contraction. Neuromuscular electrical stimulation intervention continued while the limb was immobilized in the long leg cast and following cast removal as a means of strengthening to facilitate the targeted muscle during exercises and with mobility training. After cast removal, each child attended outpatient physical therapy to continue to work on strengthening, standing balance, and improving functional mobility as part of their recovery.

### 2.3. Outcome Measures

Pre- and post-operative physical therapy assessments were completed for each child, including Functional Mobility Scale (FMS), Gross Motor Function Measures-66 (GMFM), and 6-Minute Walk Test (6MWT).

Functional Mobility Scale (FMS): The FMS is a performance measure, rating 1 to 6, to classify functional mobility at 5, 50, and 500 m to represent home, school, and community settings, respectively [15]. It is a tool to measure change in functional mobility in children with CP after orthopaedic surgery and to distinguish the ability to negotiate various surfaces (e.g., uneven ground, curbs, crowded environments) [16]. The FMS has good construct validity [15] to detect change in children with CP [15]. Inter-rater agreement has been reported as excellent (0.86 to 0.92) for all three distances [17].

Gross Motor Function Measure-66 (GMFM): The GMFM is a standardized assessment of gross motor skills for children with CP [18,19]. The GMFM is validated for children with CP across all GMFCS levels for children 5 months to 16 years old [19]. The GMFM-66 is derived from the GMFM-88 with reduced items and converted scales [20] and has excellent agreement [21]. Developmental motor curves have been established for children with CP based on age and GMFCS levels using the GMFM-66 score [18]. This measure is used for ambulatory children with CP before and after orthopaedic surgery [22].

6 Minute Walk Test (6MWT): The 6MWT is a standard measurement to assess change in function and endurance in children with CP [23,24]. Intra-rater reliability (0.8–0.88) and validity has been reported to be good [25]. Trajectories based on age and GMFCS levels have been described using the 6MWT [26].

## 3. Results

Each child tolerated NMES intervention to the quadriceps while immobilized in long leg casts using surface electrodes within the window cut-outs and did not report any adverse effects of surface electrode use or NMES intervention. Results of pre- and post-assessments for each child are summarized in Table 3.

### 3.1. FMS

The children in this case series were assessed using the FMS both pre-operatively and post-operatively (12–18 months after surgery). Child 1 had a pre-operative score of 5-5-5 and an 18-month post-operative score of 6-6-5 with an improvement from independence on level surfaces to independence on all surfaces for 5 and 50 m. Child 2 had a pre-operative score of 5-5-5 and a 12-month post-operative score of 5-5-5. While this child did not make gains in mobility, it is worth noting that the child did not decline in his scores. Child 3 had a pre-operative score of 6-5-5 and a 12-month post-operative score of 6-6-5 with an improvement from independence on all surfaces at 5 m only to independence on all surfaces at 5 and 50 m.

### 3.2. GMFM-66

All three children in this case series were assessed using the GMFM-66 Item Sets both pre-operatively and post-operatively (12–15 months after surgery). Child 1 had a pre-operative score of 65 (35th percentile for age and GMFCS level) and a 12-month post-operative score of 65.5 (30th percentile for age and GMFCS level). While this child did not make gains in gross motor skills, he did not decline in his score. Child 2 had a pre-operative score of 81.5 (80th–85th percentile for age and GMFCS level) and a 15-month post-operative score of 73.6 (55th–60th percentile for age and GMFCS level). While this child showed regression in his gross motor skills after surgery, he demonstrated expected gross motor skills (50th percentile) for his age and GMFCS level. Child 3 had a pre-operative score of 75.5 and a 12-month post-operative score of 81.9. This child demonstrated a 6.6 change in score in the GMFM-66 from pre- to post-operative assessments. This change in score was significant based on a minimally clinically important difference (MCID) in change of scores 1.5 (large effect) for individuals GMFCS level II following orthopaedic surgery [22].

### 3.3. 6MWT

In this case series, the three children were assessed using the 6MWT both pre-operatively and post-operatively (8–15 months after surgery). Child 1 had a pre-operative distance of 921 feet (25th percentile for age and GMFCS level) and a 12-month post-operative distance of 1032 feet (25th–50th percentile for age and GMFCS level). Child 2 had a pre-operative distance of 1328 feet (75th percentile for age and GMFCS level) and a 15-month post-operative distance of 1702 feet (90th–95th percentile for age and GMFCS level). Child 3 had a pre-operative distance of 1456 feet and an 8-month post-operative distance of 1817 feet. The change in distance for all three children was significant based on an MCID change score of 92 feet (estimate method) for individuals with CP GMFCS Level I-II [24].

## 4. Discussion

Neuromuscular electrical stimulation is an intervention available to physical therapists when treating children with CP. It is non-invasive and modifiable for each individual [14]. Surface electrode placement is individualized, determined by past trials, necessary to optimize NMES, and needed to facilitate motor contraction [27,28]. Although NMES is readily available, it is not broadly used as a treatment modality due to decreased knowledge, awareness of pediatric parameters, and pediatric tolerance. The physical therapists involved in the care of the children in this case series had experience with using NMES and demonstrated skill in using this intervention. Clinical proficiency has been a common theme in many studies involving NMES, especially as it relates to modifying parameters [14]. Recently, parameters have been reported and compared amongst multiple studies using NMES in children with CP [14].

Neuromuscular electrical stimulation parameters used for this case series were based on those referenced in pediatric versus adult literature [14]. Intensities of motor contraction to the quadriceps musculature using NMES has been previously reported in adults with TKA [12]. Adults with TKA were compliant and tolerated NMES immediately following their surgery [12]. In this case series, the children demonstrated the ability to participate in NMES to bilateral quadriceps while immobilized in long leg casts. In comparison to previous reports of decreased tolerance and infection from embedded electrodes [13], no adverse issues related to skin integrity were reported from use of surface electrode stimulation or lack of tolerance to the NMES intervention to the quadriceps for all three children. Neuromuscular electrical stimulation was started approximately one week following orthopaedic surgery when cut-outs were made to the casts. Previous literature reports the need for inpatient rehabilitation to assist with NMES in children with CP [13]; however, this was not needed for the children in this case series.

The outcome measures completed for this case series were based on recommendations in the literature for children with CP undergoing orthopaedic surgery [7,9,16,22,29]. Two of the three children had gains in the FMS and GMFM-66. All three children had gains in the 6MWT. The results of this case series align with reports in the literature regarding functional mobility following LE orthopaedic surgery. When compared with baseline measurements, improvements in GMFM and FMS scores would be expected by 24 months post-operatively [9,16]. Although we cannot determine if the NMES intervention facilitated each child’s rehabilitation successes in this case series, the results indicate that there were no adverse effects using surface electrodes with window cast cut-outs and expected gains were made for these three children.

The context of using NMES intervention for the children in this case series was to maintain strength or reduce muscle atrophy when the children were immobilized to promote ease of function upon cast removal. This novel technique using cast cut-outs in a long leg cast to apply surface electrodes as sensors to contract musculature while in an immobilized position fostered a new way of promoting function using NMES or functional electrical stimulation.

### 4.1. Limitations

The description of this case series represents only three children with spastic diplegia CP, GMFCS level II who participated in NMES intervention with long leg casts after SEMLS and cannot be generalized to all children with CP receiving NMES intervention to the quadriceps following orthopaedic surgery. Outcome measures and reassessment dates reported were limited to what was readily available in the clinical setting and restrictions due to COVID-19 and lengthy distances traveled by some of the children and families. In addition, we were unable to report percentiles for the GMFM-66 and 6MWT for Child 3 due to unknown developmental curves and trajectories for his age. Although the authors attempted to have a homogeneous group of children in the case series to describe, it is worth noting that Child 1 was on the lower end of the GMFCS level II developmental motor curve and Child 2 and 3 were on the upper end of the GMFCS level II developmental motor curve; as a result, this may have contributed to Child 1 having lower percentiles and Child 2 having baseline scores exceeding expectations for gross motor skills.

### 4.2. Future Research

Neuromuscular electrical stimulation should be an intervention explored with a larger number of individuals with CP following orthopaedic surgery to further investigate maintaining muscle strength and reducing muscle atrophy while immobilized in LE casts. Previous authors have reported a return to baseline mobility at 1 year following orthopaedic surgery [9]. Future efforts could determine if an even faster recovery in function could be achieved using window cast cut-outs to apply surface electrodes for NMES to targeted muscles of an immobilized limb which may ultimately reduce overall healthcare costs. Additionally, research is needed on outcome measures to assess the strength of immobilized limbs when applying NMES. One consideration for measuring strength of a targeted muscle immobilized in a cast is by means of a sensor obtaining a force measurement of an isometric muscle contracture. Research initiatives will require physical therapists having additional training to increase their comfort level in using NMES as an intervention for children with CP and clinical scientists applying sensor systems to measure strength.

## 5. Conclusions

Neuromuscular electrical stimulation intervention for quadriceps strengthening is tolerated in three children with spastic diplegia CP, GMFCS level II using surface electrodes with windowed cast cut-outs while immobilized in long leg casts following orthopaedic surgery. No adverse effects are reported and expected gains are found in functional mobility, gross motor skills, and walking endurance for the three children who received NMES intervention following SEMLS. Using NMES intervention with window cast cut-outs is a unique practice method to begin immediate rehabilitation during the immobilization period following LE orthopaedic surgery.

## Figures and Tables

**Table 1:** Child demographics and clinical characteristics.

	Child 1	Child 2	Child 3
Age at time of surgery	9 years	13 years	15 years
Gender	Male	Male	Male
Race	Asian	White	White
Type of CP	Spastic Diplegia	Spastic Diplegia	Spastic Diplegia
GMFCS Level	II	II	II
Type of Tone	Spasticity	Spasticity	Mixed (Spasticity/Dystonia)
SEMLS Procedures	Soft Tissue	Soft Tissue Tone management	Soft Tissue Tone management

**Table 2:** Neuromuscular electrical stimulation parameters.

	Child 1	Child 2	Child 3
Channel	Dual	Dual	Dual
Intensity/Pulse Amplitude	9.75 mA	17 mA	26–32.5 mA
Ramp Time	2 s	1 s	2 s
On:Off Time	10:30 s	5:10 s	5:10 s
Pulse Rate/Frequency	35 Hz	35 Hz	35 Hz
Pulse Width/Pulse Duration	350 µs	200 µs	200 µs
Output Waveform	Symmetrical, Biphasic	Symmetrical, Biphasic	Symmetrical, Biphasic
Output Mode	Synchronous	Synchronous	Synchronous
Targeted Muscle Groups	Bilateral Quadriceps	Bilateral Quadriceps	Bilateral Quadriceps
Electrode Size	2 × 2 square	2 × 2 square	2 × 2 square
Treatment time	15–20 min	15–20 min	30 min

Legend: mA = milliamps, V = Volts, Hz = Hertz, µs = microseconds.

**Table 3:** Pre- and post-operative outcome measures following NMES intervention in children undergoing lower extremity orthopaedic surgery.

	Child 1	Child 2	Child 3
**Pre-operative**			
GMFM score	65	81.5	75.3
FMS score	5-5-5	5-5-5	6-5-5
6-Min Walk Test distance	921 feet	1328 feet	1456 feet
**Post-operative**			
GMFM score	65.5	73.6	81.9
FMS score	6-6-5	5-5-5	6-6-5
6-Min Walk Test distance	1032 feet	1702 feet	1817 feet
**Change Pre- to Post-operatively**			
GMFM point change	0.5	−7.9	6.6 *
FMS rating change in meters (m)	Increase 5 m and 50 m	No change	Increase 50 m
6-Min Walk Test distance	111 feet *	374 feet *	361 feet *

* Greater than the minimally clinically important difference.

## Data Availability

Data for this case series are reported in full in the manuscript.
